# The effect of worker clothing color on stress in laying hens

**DOI:** 10.5194/aab-67-145-2024

**Published:** 2024-04-05

**Authors:** Murat Genc, Ugur Ozenturk

**Affiliations:** Department of Animal Science, Faculty of Veterinary Medicine, Atatürk University, 25170 Erzurum, Türkiye

## Abstract

The environment in which animals are kept must provide suitable conditions for their species. This includes ensuring that animals are healthy, well-fed, safe, able to exhibit species-specific behaviors, not experiencing fear or pain, and not under chronic or acute stress. Poultry welfare is achieved when birds are raised in environments that meet their physiological and ethological needs. Fear can significantly impact animal welfare. Chickens have been significantly altered by human artificial selection. Despite this, they exhibit reactivity towards humans and tend to avoid them. Poultry animals reared in environmentally controlled poultry houses and bred for superior productivity are more sensitive to fear factors and have lost their adaptability to a great extent. This study aimed to determine the effect of personnel clothing color on stress and fear in chickens in layer hen coops. The experiment involved 32-week-old laying hens of three different genotypes. A worker in the henhouse wore six respective different colors of workwear (dark blue, green, red, yellow, black, and white), and sound measurements were taken during this time. The results showed that the color of the worker's clothing influenced the sound intensity of the chickens ( P<0.05 ). White clothing elicited the least reaction, whereas black and dark blue elicited the most. The other three colors showed similar reactions. In conclusion, workers in layer hen coops wearing dark clothing, such as dark blue and black, can induce stress and noise in the animals. Additionally, chickens showed similar reactions to green, red, and yellow colors, with white being the color around which they felt the most secure.

## 1 Introduction

Animal welfare refers to the state of physical and emotional well-being of animals. It is a multifaceted subject encompassing scientific, ethical, economic, cultural, social, religious, and political dimensions. Animals raised in environments that do not prioritize welfare often experience increased mortality due to stress and stress-related diseases (Bousfield and Brown, 2010). Stress is characterized by the responses animals exhibit to adapt or protect themselves from the adverse effects of stressors (Qi et al., 2017). Factors such as extreme temperatures, exposure to toxins (especially mycotoxins) through feed, poor husbandry conditions (such as overcrowding, transportation errors, and feeding issues), certain infections, and exposure to chemicals are known to be significant stressors (El-Lethey et al., 2000). Chickens in commercial egg-laying units experience numerous stressors during breeding and rearing. These include acute or chronic factors such as sex sorting, vaccination, transport after hatching, absence of maternal care, crowding, thwarting of various behavioral needs, and mixing with unfamiliar conspecifics. Additionally, environmental factors like ammonia emissions and other climate parameters can further contribute to their stress (Appleby et al., 2004; Bist et al., 2023; Cronin et al., 2020; Hedlund and Jensen, 2021; Hofmann et al., 2020; Li et al., 2020; Ozenturk and Yildiz, 2021; Van Poucke et al., 2023). In other words, chickens experience varying degrees of both psychological and physiological stress at different stages of their lives. Providing animals with ideal housing conditions and managing their exposure to stressors properly can positively affect their productivity and welfare (Chrousos, 1997; Sapolsky, 1999).

Stress in poultry primarily leads to disruptions in health and productivity, although it can also result in behavioral disorders such as cannibalism and feather pecking (Cronin et al., 2018; Michel et al., 2022; Ozenturk et al., 2023). When birds are under stress, their blood glucose levels increase, depleting glycogen, the storage sugar in the liver and muscles. This leads to irregular respiratory rates. Stress hormones also disrupt the balance of intestinal microflora, altering pH levels in the intestines and creating an environment conducive to the reproduction of various bacteria and fungi, potentially leading to gastrointestinal diseases. Furthermore, stress in poultry depresses the immune system, reducing production and increasing the rates of culls and deaths. Suppressed immune systems make birds more susceptible to viral, bacterial, protozoan, and fungal infections, leading to stress syndrome. Additionally, stress causes a decrease in metabolic functions (Rosales, 1994; Ognik and Sembratowicz, 2012)

Stress in poultry is challenging to determine due to its dependence on various factors. Typically, four parameters – health, productivity, behavior, and physiology – are employed to assess stress in poultry species (Chrousos, 1997; Sapolsky et al., 2000). Commonly used stress markers include the circulating heterophile / lymphocyte ratio, adrenocortical hormone and corticosterone levels, and adrenal gland weight. However, the analysis of these stress markers requires specialized expertise, and the methods are often costly and labor-intensive (Nwaigwe et al., 2020; Lee et al., 2022). Nevertheless, some stress determination methods are more cost-effective and require less labor.

The growing interaction between humans and animals has sparked greater curiosity about animals' lives and behaviors, leading to the inclusion of behavior patterns in the realm of scientific inquiry (Akbaş, 2013). Recent focus has shifted towards evaluating animal welfare and stress levels through an examination of their emotional states (Vasdal et al., 2018). While an animal's emotional state cannot be directly measured, it is typically assessed through physiological and behavioral factors (Wemelsfelder and Lawrence, 2001; McMillan, 2020).

Various tests exist to measure fear-related stress in poultry, such as the avoidance distance test, touch test, novel object test, and stationary person test (Brantsæter et al., 2017; Rasmussen et al., 2022; Vasdal et al., 2022). One method involves measuring the sounds that animals produce (Ríos-Chelén et al., 2017). Hens, for example, may vocalize in response to fear or stress, making sounds such as clucking, or squealing at different frequencies and intensities (Collias, 1987; Neethirajan, 2023). The intensity and frequency of these vocalizations can vary based on the perceived threat and the individual temperament of the bird. Additionally, laying hens may exhibit increased movement when experiencing fear or stress, which can include flapping wings, pacing, or other agitated behaviors. This heightened activity often serves as a natural response to perceived threats, as birds may attempt to escape or avoid the source of stress. Increased movement within a cage or enclosure can contribute to higher noise levels (Nicol, 2015).

Auditory, olfactory, visual, or physical contact of birds with humans can induce fear. Poultry are particularly sensitive to visual stimuli, including colors, and may exhibit different reactions to various hues. Research into animal behavior and welfare has indicated that certain colors might have calming or stressing effects on animals (Hesham et al., 2018). The presence of individuals other than the farmer or a change in the farmer's attire upon entering the poultry farm may elicit fear and panic (Kilgour and Dalton, 1984; Jones, 1987). According to a study, layer hens raised in indoor poultry farms can detect an approaching object from a distance of 25 m (Jones et al., 1981).

Chickens possess remarkable color vision and can perceive a wide range of color spectra, including violet and ultraviolet (UV) light. They are capable of seeing UV-A light (315–400 nm) in addition to the visible spectrum of 400–750 nm. In addition to their adeptness at perceiving colors, chickens have a broader visual capability than previously understood (Ham and Osorio, 2007; Olsson et al., 2015).

Chickens have the same three primary color cones (red, yellow, and blue) as humans, but they also possess a UV cone. This additional cone enables chickens to perceive colors and shades beyond human perception, allowing them to distinguish and perceive a broader range of colors and tones. Furthermore, chickens exhibit exceptional focusing abilities, enabling them to visually perceive objects both near and at a distance (Seifert et al., 2020). From an early age, chickens demonstrate good eyesight. Shortly after hatching, chicks are capable of navigating around obstacles, detecting moving objects, and pecking objects with precision. They can also differentiate between shallow and deep surfaces (Ham and Osorio, 2007; Seifert et al., 2020).

To ensure sustainability, production efficiency, and the health and welfare of animals, a comprehensive management program is essential from hatching to the end of production. Every stress factor that may occur during the growing and yield period is associated with a reduction in the welfare and productivity of the animals. Furthermore, individuals working in the coop can impact the health, welfare, and performance of the animals. Additionally, unfavorable housing conditions can have adverse effects on the health and well-being of both animals and workers. This study aimed to investigate the impact of color differences in worker clothing on the stress levels of animals in laying hen coops.

## 2 Materials and methods

The research was conducted at the Food and Animal Farming Research and Application Center of Atatürk University and was prepared in accordance with the Declaration of Helsinki and animal welfare guidelines. Three different genotypes were used in the experiment: one domestic (Atak-S) and two foreign (ISA Brown and Novogen White). All animals were 32 weeks old. The Atak-S genotype is a recent addition to Türkiye's breeding studies (Ozenturk and Yildiz, 2020; Akunal and Koknaroglu, 2021). In Turkey, domestic layer hybrids were produced at the Ankara Poultry Research Institute in 1995 using pure lines imported from Canada (Fathel and Elibol, 2006). The ISA Brown (IB), Novogen White (NW), and Atak-S (AS) hybrids have brown, white, and black plumage colors, respectively. IB and AS hybrids lay brown eggs, whereas NW hybrids lay white eggs (Table 1). It has been reported that AS hybrids achieve annual egg yields ranging from 75 % to 80 % (280–300 eggs) and foreign hybrids achieve yields ranging from 80 % to 85 % (300–320 eggs) within the 72-week age period (Turker et al., 2017; Ozenturk and Yildiz, 2021).

**Table 1:** Animals in the experiment.

Hybrid	Egg	Plumage	Origin	Number
	color	color		
Atak-S	Brown	Black	Domestic	1080
Novogen White	White	White	Foreign	1080
ISA Brown	Brown	Brown	Foreign	1080

### 2.1 Cage design

The henhouse consists of three blocks, each containing three tiers. Cage compartments are symmetrically arranged on both sides (left and right) of each block, with each tier comprising 60 cage compartments (30 on each side). Six birds were housed in each cage compartment, and all hybrids (IB, AS, and NW) were evenly distributed among the cages to ensure uniformity.

The chickens were housed in multistory cages within a single building with windows, with six chickens per cage and a floor area of 625 cm ${}^{2}$ per chicken. To provide uniform illumination, the lamps in the henhouses were positioned at equal distances from each other. Each cage compartment was 60 cm deep, 62.5 cm wide, 46 cm high at the rear, and 51 cm high at the front with a 62.5 cm feeder length and a 7° base tilt. Each cage was equipped with two nipple drinkers within the reach of each bird.

The in-house temperature was maintained between 16 and 24 °C by a sensor connected to the ventilation and heating system. The poultry house has windows for natural daylight. A lighting program of 16 h d ${}^{-1}$ was implemented using fluorescent lamps (4000 K) that provide white light. During the first 4 weeks of the laying period (weeks 16–20), the hens were fed egg starter feed (2750 ME (metabolic energy), 17.50 % CP (crude protein)), followed by laying feed (2750 ME, 16.26 % CP) in granule form ad libitum from the 21st to the 45th week.

### 2.2 Sound measurement method

The worker responsible for the care of the chickens wore six respective different colors of workwear (dark blue, green, red, yellow, black, and white) and walked around the same route in the poultry house for 5 min in each set of clothes. The worker did not have a fixed outfit that they wore regularly every day. The entrance and exit to the poultry house were controlled during the experiment, ensuring that no other personnel entered the house both during the trial and at other times. The worker refrained from making visual or tactile contact with the chickens while walking around.

Sound measurements were taken after the first 3 min to allow the animals to adapt to a new clothing color. The measurements were taken after the worker had completed one round through all of the cages. While the worker was walking around in different respective workwear colors, sound measurements were continuously recorded for 2 min using a Trotec SL300^®^ device and captured on video. The sound meter was positioned centrally in the middle of the house. A 10 min break was provided between the two sets of color measurements to allow the chickens to calm down. Sound measurements were taken at 09:00 and 21:00 LT (local time) for 10 consecutive days.

### 2.3 Statistical analysis

Decibel (dB) values were determined every 10 s in the 2 min videos in which the sound values were recorded. The IBM SPSS software package was used in the analysis of the data obtained in the experiment. Statistical analyses were performed using every 10 s data point as repeated measurements. The GLM (general linear model) repeated-measures procedure was applied to determine the effect of color, time, and day vs. night on the sound of chickens, and the results are given as the mean ± SE (standard error). Interactions were removed from the statistical model because they were insignificant, and only the main affects were examined.

## 3 Results

**Figure 1:**
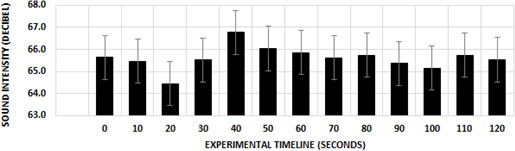
Effect of the experimental timeline on sound intensity.

Based on the findings of this work, it was determined that the sound intensity of the chickens did not change according to the experimental timeline ( F(12444)=0.423 , P=0.954 ). There was no statistically significant difference between the values recorded every 10 s during the 120 s measurements (Fig. 1).

The chickens exhibited a greater noise reaction to the worker's clothing color differences in the morning compared with the evening. The average sound intensity during the daytime measurements was 66.22 ± 0.32 dB, whereas it was 64.99 ± 0.32 dB during nighttime measurements. A significant difference was found between the sound measurements taken in the morning and evening ( F(1444)=7.532 , P=0.006 ).

**Figure 2:**
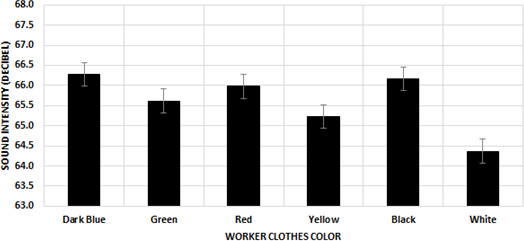
Effect of the worker's clothing color on the mean sound intensity.

It has been determined that the color of the worker's clothing affects the sound intensity of the chickens ( F(5444)=2.598 , P=0.048 ). It was determined that the color to which the chickens reacted the least was white (64.37 ± 0.55 dB), whereas the colors to which they reacted the most were black (66.17 ± 0.56 dB) and dark blue (66.28 ± 0.54 dB). It was observed that similar values were obtained for the other three workwear clothing colors (Fig. 2). These values were measured as 65.62 ± 0.54, 65.98 ± 0.54, and 65.23 ± 0.54 dB for green, red, and yellow, respectively.

## 4 Discussion

Animals experience varying degrees of both psychological and physiological stress at different stages of their lives. To prevent overexposure to these stress factors, it is important to identify the factors causing stress. Managing an animal's exposure to stress factors correctly can not only positively affect their productivity but also increase their welfare (Chrousos, 1997; Sapolsky, 1999; Ozenturk and Yildiz, 2021; Ozenturk et al., 2023).

Ongoing research on laying hens has demonstrated that bird vocalizations can serve as reliable indicators of particular forms of stress and that different vocalization patterns can be linked with specific kinds of stressful conditions (Neethirajan, 2023). Research findings indicate that chickens exhibit a statistically higher noise response in the morning compared with the evening. Chickens, like many other bird species, are diurnal, meaning they are active during the day and rest at night (Deep et al., 2012). Thus, the noise difference may be explained by the fact that chickens are active and noisy during the day but generally roost and sleep at night.

Less noise was detected when workers wore white clothing in the chicken coop compared with other colors. Dark blue and black colors elicited the loudest noise, while red, green, and yellow colors resulted in medium noise levels. White is a bright and highly visible color to birds, creating a high contrast against the surroundings. In contrast, dark colors like black and dark blue may not be as easily visible, potentially making movements or objects more challenging for laying hens to detect. This increased visibility of personnel in white clothing may make their presence more predictable and less startling to the birds (Meuser et al., 2021). Furthermore, in nature, birds often associate dark colors with potential predators. Predatory birds and animals that threaten laying hens may have darker plumage or fur, leading to an innate fear response to dark colors. White, being a less common color in predators, may not trigger the same fear response (Hughes, 1997; Meuser et al., 2021).

Chickens have the ability to produce sounds ranging from approximately 60 to 80 decibels (dB), with the actual output dependent on the context and the individual chicken (Donofre et al., 2020; Ginovart-Panisello et al., 2020; Hill et al., 2014; Nicol, 2015). This range of vocalizations plays a crucial role in their communication and social interactions. For instance, in response to potential threats, chickens can exhibit a notable difference in vocalization volume between a calm state and a panicked state, serving as a natural alarm response (Brouček, 2014; Campo et al., 2005; Laurijs et al., 2021; Nicol, 2015). In our study, we found a statistically significant difference of approximately 2 dB in sound levels between trials in which chickens were exposed to white clothing (64.37 dB) and trials in which they were exposed to dark clothing (black: 66.17 dB; dark blue: 66.28 dB). While this difference may seem minor, it can be meaningful in specific contexts, particularly when considering the sensitivity of sound measurements and the animals' perception of sound. Chickens, like other animals, may exhibit heightened sensitivity to changes in sound levels compared with humans (Brouček, 2014; Hill et al., 2014; Olczak et al., 2023; Tefera, 2012). A 2 dB increase could indicate a noticeable change in their environment, potentially signaling a shift in behavior or alertness. Furthermore, even slight increases in sound levels over time can have a cumulative effect on animals, potentially leading to stress or other behavioral changes (Brouček, 2014; Campbell et al., 2019; Campo et al., 2005; Nielsen et al., 2023; Zhao et al., 2023). Therefore, these findings have important implications for the welfare and management of poultry.

To our knowledge, there is no literature similar to this study, which was conducted to determine the effect of worker clothing color on stress in chickens. However, there are studies on different light and equipment colors. For example, one study found that white and red light made chickens more active, but red light caused aggression. Additionally, blue or green light kept the chickens calmer (Prayitno, 1997). Another study reported that chickens raised in red light showed more activity and aggressive behavior (Hesham et al., 2018). Similarly, red light (700 nm) was found to increase the activity and aggressive behavior of chickens (Soliman and El-Sabrout, 2020). In a study on broilers with four different light colors (red, blue, white, and green), animals preferred white (Rierson, 2011). This result is similar to the findings of the present study. Another study emphasized that white light had a positive effect on increasing feed and water consumption by stimulating activity in layer chicks. It was also noted that red is an attractive color for chickens; therefore, red drinkers and feeders are generally used in poultry houses (Fidan and Nazligul, 2005). In this study, the excessive reaction of chickens to the color black may explain why they do not prefer this color. In another study using feeds of different colors, chickens especially preferred red (Forbes and Covasa, 1995), indicating that color is a strong cue for chickens in learning about preference and aversion (Jones et al., 2000).

Further research is necessary to determine the exact threshold for perceivable differences in loudness for chickens across various contexts. Such investigations can provide valuable insights into how chickens perceive and respond to changes in their environment, aiding in the development of effective management strategies.

## 5 Conclusions

In summary, it was determined that laying hens react more to dark colored work clothes such as dark blue and black, causing increased noise in the poultry house. This may have been due to the fact that dark colors caused stress in the animals. It was also found that animals showed similar reactions to green, red, and yellow colors, with white resulting in the least noise. Based on these results, it is thought that personnel wearing white clothes when working in poultry houses would be beneficial not only for animal welfare and health but also for their own health, as chronic noise can lead to various health problems, especially hearing problems. It is recommended that the effect of workwear colors on noise be examined by testing different workwear colors in different chicken coops. Additionally, the scientific understanding of color perception and its impact on poultry behavior is an evolving field, and more research may provide additional insights into this aspect of poultry management.

## Data Availability

The data used and analyzed during this study are available from the corresponding author upon reasonable request.

## References

[bib1.bib1] Akbaş A (2013). Farm animal behaviour and welfare relationship. Mehmet Akif Ersoy University Journal of Health Sciences Institute.

[bib1.bib2] Akunal T, Koknaroglu H (2021). Commercial native laying hybrids developed in Turkey are comparable to foreign hybrids in terms of performance and cultural energy use efficiency. Anim Sci Pap Rep.

[bib1.bib3] Appleby MC, Mench JA, Hughes BO (2004). Poultry behaviour and welfare.

[bib1.bib4] Bist RB, Subedi S, Chai L, Yang X (2023). Ammonia emissions, impacts, and mitigation strategies for poultry production: A critical review. J Environ Manage.

[bib1.bib5] Bousfield B, Brown R (2010). Animal welfare. Vet Bull.

[bib1.bib6] Brantsæter M, Tahamtani FM, Nordgreen J, Sandberg E, Hansen TB, Rodenburg TB, Moe RO, Janczak AM (2017). Access to litter during rearing and environmental enrichment during production reduce fearfulness in adult laying hens. Appl Anim Behav Sci.

[bib1.bib7] Brouček J (2014). Effect of noise on performance, stress, and behaviour of animals. Slovak J Anim Sci.

[bib1.bib8] Campbell DLM, De Haas EN, Lee C (2019). A review of environmental enrichment for laying hens during rearing in relation to their behavioral and physiological development. Poult Sci.

[bib1.bib9] Campo JL, Gil MG, Davila SG (2005). Effects of specific noise and music stimuli on stress and fear levels of laying hens of several breeds. Appl Anim Behav Sci.

[bib1.bib10] Chrousos GP (1997). Stressors, stress, and neuroendocrine integration of the adaptive response. Ann N Y Acad Sci.

[bib1.bib11] Collias NE (1987). The vocal repertoire of the red junglefowl: A spectrographic classification and the code of communication. Condor.

[bib1.bib12] Cronin GM, Hopcroft RL, Groves PJ, Hall EJS, Phalen DN, Hemsworth PH (2018). Why did severe feather pecking and cannibalism outbreaks occur? An unintended case study while investigating the effects of forage and stress on pullets during rearing. Poult Sci.

[bib1.bib13] Cronin GM, Glatz PC, Cronin GM, Glatz PC (2020). Causes of feather pecking and subsequent welfare issues for the laying hen: A review. Anim Prod Sci.

[bib1.bib14] Deep A, Schwean-Lardner K, Crowe TG, Fancher BI, Classen HL (2012). Effect of light intensity on broiler behaviour and diurnal rhythms. Appl Anim Behav Sci.

[bib1.bib15] Donofre AC, Da Silva IJO, Ferreira IEP (2020). Sound exposure and its beneficial effects on embryonic growth and hatching of broiler chicks. Br Poult Sci.

[bib1.bib16] El-Lethey H, Aerni V, Jungi TW, Wechsler B (2000). Stress and feather pecking in laying hens in relation to housing conditions. Br Poult Sci.

[bib1.bib17] Fathel AN, Elibol O (2006). Comparison of domestic and foreign brown layer hybrids in terms of productivity characteristics. J Agr Sci.

[bib1.bib18] Fidan ED, Nazligul A (2005). The effects of light source on the Japanese quails (Coturnix coturnix japonica) some production traits. Kocatepe Vet J.

[bib1.bib19] Forbes JM, Covasa M (1995). Aplication of diet selection by poultry with particular reference to whole cereals. Worlds Poult Sci J.

[bib1.bib20] Ginovart-Panisello GJ, Alsina-Pagès RM, Sanz II, Monjo TP, Prat MC (2020). Acoustic description of the soundscape of a real-life intensive farm and its impact on animal welfare: A preliminary analysis of farm sounds and bird vocalisations. Sensors.

[bib1.bib21] Ham AD, Osorio D (2007). Colour preferences and colour vision in poultry chicks. Proc Biol Sci.

[bib1.bib22] Hedlund L, Jensen P (2021). Incubation and hatching conditions of laying hen chicks explain a large part of the stress effects from commercial large-scale hatcheries. Poult Sci.

[bib1.bib23] Hesham MH, El Shereen AH, Enas SN (2018). Impact of different light colors in behavior, welfare parameters and growth performance of Fayoumi broiler chickens strain. J Hell Vet Med Soc.

[bib1.bib24] Hill EM, Koay G, Heffner RS, Heffner HE (2014). Audiogram of the chicken (Gallus gallus domesticus) from 2 Hz to 9 kHz. J Comp Physiol A.

[bib1.bib25] Hofmann T, Schmucker SS, Bessei W, Grashorn M, Stefanski V (2020). Impact of housing environment on the immune system in chickens: A review. Animals.

[bib1.bib26] Hughes RN (1997). Intrinsic exploration in animals: motives and measurement. Behav Process.

[bib1.bib27] Jones RB, Zayan R, Duncan IJH (1987). Cognitive Aspects of Social Behaviour in the Domestic Fowl.

[bib1.bib28] Jones RB, Duncan IJH, Hughes BO (1981). The assessment of fear in domestic hens exposed to a looming human stimulus. Behav Process.

[bib1.bib29] Jones RB, Carmichael NL, Rayner E (2000). Pecking preferences and pre-dispositions in domestic chicks: Implications for the development of environmental enrichment devices. Appl Anim Behav Sci.

[bib1.bib30] Kilgour R, Dalton C (1984). Livestock Behaviour.

[bib1.bib31] Laurijs KA, Briefer EF, Reimert I, Webb LE (2021). Vocalisations in farm animals: A step towards positive welfare assessment. Appl Anim Behav Sci.

[bib1.bib32] Lee C, Kim JH, Kil DY (2022). Comparison of stress biomarkers in laying hens raised under a long-term multiple stress condition. Poult Sci.

[bib1.bib33] Li D, Tong Q, Shi Z, Li H, Wang Y, Li B, Yan G, Chen H, Zheng W (2020). Effects of chronic heat stress and ammonia concentration on blood parameters of laying hens. Poult Sci.

[bib1.bib34] McMillan FD, McMillan FD (2020). Mental Health and Well-Being in Animals.

[bib1.bib35] Meuser V, Weinhold L, Hillemacher S, Tiemann I (2021). Welfare-related behaviors in chickens: Characterization of fear and exploration in local and commercial chicken strains. Animals.

[bib1.bib36] Neethirajan S (2023). Vocalization Patterns in Laying Hens-An Analysis of Stress-Induced Audio Responses. bioRxiv.

[bib1.bib37] Nicol CJ (2015). The behavioural biology of chickens.

[bib1.bib38] Nielsen SS, Alvarez J, Bicout DJ, Calistri P, Canali E, Drewe JA, Garin-Bastuji B, Rojas JLG, Schmidt CG, Herskin M, Michel V, Chueca MAM, Padalino B, Pasquali P, Roberts HC, Spoolder H, Stahl K, Velarde A, Viltrop A, Winckler C, EFSA Panel on Animal Health and Animal Welfare (AHAW) (2023). Welfare of laying hens on farm. EFSA J.

[bib1.bib39] Nwaigwe CU, Ihedioha JI, Shoyinka SV, Nwaigwe CO (2020). Evaluation of the hematological and clinical biochemical markers of stress in broiler chickens. Vet World.

[bib1.bib40] Michel V, Berk J, Bozakova N, van Der Eijk J, Estevez I, Mircheva T, Relic R, Rodenburg TB, Sossidou EN, Guinebretière M (2022). The relationships between damaging behaviours and health in laying hens. Animals.

[bib1.bib41] Ognik K, Sembratowicz I (2012). Stress as a factor modifying the metabolism in poultry – A review. Annales UMCS, Zootechnica.

[bib1.bib42] Olczak K, Penar W, Nowicki J, Magiera A, Klocek C (2023). The Role of Sound in Livestock Farming-Selected Aspects. Animals.

[bib1.bib43] Olsson P, Lind O, Kelber A (2015). Bird colour vision: behavioural thresholds reveal receptor noise. J Exp Biol.

[bib1.bib44] Ozenturk U, Yildiz A (2020). Assessment of egg quality in native and foreign laying hybrids reared in different cage densities. Braz J Poult Sci.

[bib1.bib45] Ozenturk U, Yildiz A (2021). Comparison of performance parameters, stress, and immunity levels of native andcommercial layers reared in different cage densities in Turkey. Turkish J Vet Anim Sci.

[bib1.bib46] Ozenturk U, Yildiz A, Genc M (2023). Assessment of the feather score and health score in laying hens reared at different cage densities. Vet J Ankara Univ.

[bib1.bib47] Prayitno DS, Phillips CJ, Omed H (1997). The effects of color of lighting on the behavior and production of meat chickens. Poult Sci.

[bib1.bib48] Ríos-Chelén AA, McDonald AN, Berger A, Perry AC, Krakauer AH, Patricelli GL (2017). Do birds vocalize at higher pitch in noise, or is it a matter of measurement?. Behav Ecol Sociobiol.

[bib1.bib49] Qi J, Zhang Y, Zhou Z, Habiba U (2017). Parameters of physiological responses and meat quality in poultry subjected to transport stress. Biol Syst Open Access.

[bib1.bib50] Rasmussen SN, Erasmus M, Riber AB (2022). The relationships between age, fear responses, and walking ability of broiler chickens. Appl Anim Behav Sci.

[bib1.bib51] Rierson RD (2011). Broiler preference for light color and feed form, and the effect of light on growth and performance of broiler chicks [phd thesis].

[bib1.bib52] Ríos-Chelén AA, McDonald AN, Berger A, Perry AC, Krakauer AH, Patricelli GL (2017). Do birds vocalize at higher pitch in noise, or is it a matter of measurement?. Behav Ecol Sociobiol.

[bib1.bib53] Rosales AG (1994). Managing stress in broiler breeders: A review. J Appl Poult Res.

[bib1.bib54] Sapolsky RM (1999). Glucocorticoids, stress, and their adverse neurological effects: relevance to aging. Exp Gerontol.

[bib1.bib55] Sapolsky RM, Romero LM, Munck AU (2000). How do glucocorticoids influence stress responses? Integrating permissive, suppressive, stimulatory, and preparative actions. Endocr Rev.

[bib1.bib56] Seifert M, Baden T, Osorio D (2020). The retinal basis of vision in chicken. Semin Cell Dev Biol.

[bib1.bib57] Soliman FN, El-Sabrout K (2020). Light wavelengths/colors: Future prospects for broiler behavior and production. J Vet Behav.

[bib1.bib58] Tefera M (2012). Acoustic signals in domestic chicken (*Gallus gallus*): A tool for teaching veterinary ethology and implication for language learning. Ethiop Vet J.

[bib1.bib59] Turker I, Alkan S, Akcay S (2017). Comparison of domestic and foreign commercial brown layer hens in terms of yield characteristics in free-range raising system. Turkish JAF Sci Tech.

[bib1.bib60] Van Poucke E, Suchánková H, Jensen P (2023). Commercial hatchery processing may affect susceptibility to stress in laying hens. Plos One.

[bib1.bib61] Vasdal G, Moe RO, de Jong IC, Granquist EG (2018). The relationship between measures of fear of humans and lameness in broiler chicken flocks. Animal.

[bib1.bib62] Vasdal G, Muri K, Stubsjøen SM, Moe RO, Kittelsen K (2022). Qualitative behaviour assessment as part of a welfare assessment in flocks of laying hens. Appl Anim Behav Sci.

[bib1.bib63] Wemelsfelder F, Lawrence AB (2001). Qualitative assessment of animal behaviour as an on-farm welfare-monitoring tool. Acta Agriculturae Scandinavica, A.

[bib1.bib64] Zhao S, Cui W, Yin G, Wei H, Li J, Bao J (2023). Effects of Different Auditory Environments on Behavior, Learning Ability, and Fearfulness in 4-Week-Old Laying Hen Chicks. Animals.

